# Supplementary figures and images for: Effect of wall type, delayed mortality and mosquito age on the residual efficacy of a clothianidin-based indoor residual spray formulation (SumiShield™ 50WG) in southern Mozambique
Source: PLoS One. 2021 Aug 5;16(8):e0248604. doi: 10.1371/journal.pone.0248604 (PMC8341595; doi:10.1371/journal.pone.0248604)

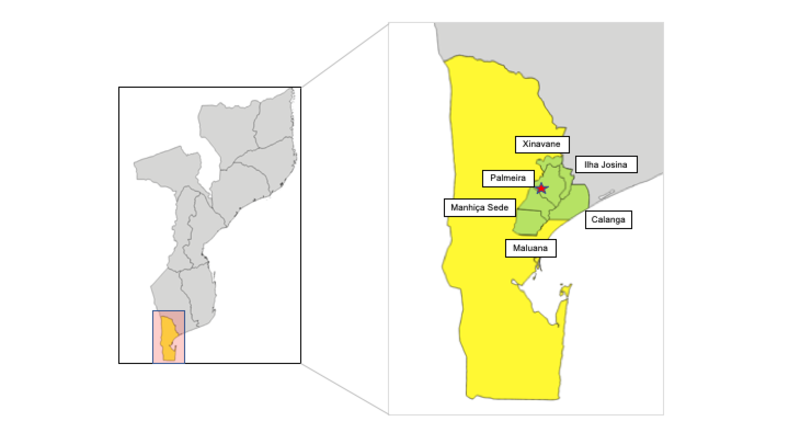

Supplement: S1 Fig — (TIF) [file pone.0248604.s001.tif]
