# Supplementary material for: Effect of wall type, delayed mortality and mosquito age on the residual efficacy of a clothianidin-based indoor residual spray formulation (SumiShield™ 50WG) in southern Mozambique
Source: PLoS One. 2021 Aug 5;16(8):e0248604. doi: 10.1371/journal.pone.0248604 (PMC8341595; doi:10.1371/journal.pone.0248604)
Supplement: S1 Table — Percentage indicates percent mortality 24h following 1h exposure in the WHO tube assay; number between parentheses indicates the number of mosquitoes tested. (DOCX) [file pone.0248604.s006.docx]

**S1 Table.** **Insecticide susceptibility of the *An. arabiensis* KGB colony maintained at the Manhiça Health Research Centre.** Percentage indicates percent mortality 24h following 1h exposure in the WHO tube assay; number between parentheses indicates the number of mosquitoes tested.

|  |  | **Percent mortality (n)** | |
| --- | --- | --- | --- |
| **Insecticide** | **Test Date** | **Treated** | **Control** |
| DDT 4% (test 1) | 23/04/2019 | 96% (100) | 2% (50) |
| DDT 4% (test 2) | 12/05/2019 | 100% (100) | 2% (50) |
| Deltamethrin 0.05% | 11/04/2019 | 100% (101) | 6% (50) |
| Pirimiphos-methyl 0.25% | 22/03/2019 | 100% (98) | 0% (50) |
| Bendiocarb 0.1% | 02/05/2019 | 100% (100) | 0% (50) |
