# Supplementary material for: Effect of wall type, delayed mortality and mosquito age on the residual efficacy of a clothianidin-based indoor residual spray formulation (SumiShield™ 50WG) in southern Mozambique
Source: PLoS One. 2021 Aug 5;16(8):e0248604. doi: 10.1371/journal.pone.0248604 (PMC8341595; doi:10.1371/journal.pone.0248604)
Supplement: S2 Table — A total of 30 mosquitoes are exposed per house, 10 mosquitoes at each height on the wall, for 30 minutes. The results shown are aggregated across these bioassays and adjusted for control mortality (10 mosquitoes at each wall height tested on unsprayed surfaces). (DOCX) [file pone.0248604.s007.docx]

**S2 Table.** **Percent monthly mosquito mortality 24 to 120h post-exposure to SumiShield^TM^ 50WG on cement and mud-plastered walls in southern Mozambique, using young (2-5d old) susceptible *An. arabiensis* females**. A total of 30 mosquitoes are exposed per house, 10 mosquitoes at each height on the wall, for 30 minutes. The results shown are aggregated across these bioassays and adjusted for control mortality (10 mosquitoes at each wall height tested on unsprayed surfaces).

|  | Cement | | | | | | | Mud | | | | |  |
| --- | --- | --- | --- | --- | --- | --- | --- | --- | --- | --- | --- | --- | --- |
|  |  | Time post-exposure | | | | | |  | Time post-exposure | | | | |
| Month | # houses | | 24h | 48h | 72h | 96h | 120h | # houses | 24h | 48h | 72h | 96h | 120h |
| 1.5 | 7 | | 65 | 79 | **87** | **88** | **89** | 9 | 65 | **87** | **98** | **98** | **99** |
| 2.5 | 4 | | 38 | 61 | 73 | **85** | **88** | 3 | 42 | 63 | **84** | **100** | **100** |
| 3.5 | 6 | | 47 | 62 | **81** | **98** | **100** | 6 | 25 | 61 | 79 | **96** | **100** |
| 4.5 | 6 | | 53 | 68 | **81** | **100** | **100** | 6 | 21 | 36 | 49 | 55 | **86** |
| 5.5 | 6 | | 51 | **96** | **99** | **99** | **100** | 6 | 24 | 60 | 75 | 79 | **86** |
| 6.5 | 6 | | 56 | 69 | **83** | **93** | **97** | 6 | 33 | 56 | **88** | **96** | **100** |
| 7.5 | 3 | | 24 | 52 | 73 | **96** | **99** | 4 | 51 | 74 | **89** | **95** | **97** |
| 8.5 | 9 | | 37 | 49 | 70 | **83** | **91** | 8 | 18 | 43 | 58 | 78 | **86** |
| 9.5 | 6 | | 14 | 32 | 73 | **81** | **95** | 6 | 31 | 51 | 64 | **81** | **93** |
| 10.5 | 6 | | 18 | 36 | 43 | 69 | 70 | 6 | 24 | 27 | 56 | 67 | 73 |
| 11.5 | 6 | | 11 | 10 | 19 | 31 | 48 | 6 | 20 | 28 | 49 | 57 | 70 |
| 12.5 | 6 | | 10 | 8 | 22 | 41 | 55 | 6 | 4 | 17 | 27 | 36 | 54 |
