# Supplementary material for: Effect of wall type, delayed mortality and mosquito age on the residual efficacy of a clothianidin-based indoor residual spray formulation (SumiShield™ 50WG) in southern Mozambique
Source: PLoS One. 2021 Aug 5;16(8):e0248604. doi: 10.1371/journal.pone.0248604 (PMC8341595; doi:10.1371/journal.pone.0248604)
Supplement: S4 Table — Lethal times are given in months post-exposure. (DOCX) [file pone.0248604.s009.docx]

**S4 Table. Estimated lethal times for 90%, 50% and 10% mortalities (LT90, LT50 and LT10, respectively) of young (2-5d old) and older (13-26d) mosquitoes** **24 to 120h post-exposure to SumiShield^TM^ 50WG on cement and mud -plastered walls in southern Mozambique**. Lethal times are given in months post-exposure.

|  |  | Young | | Old | |
| --- | --- | --- | --- | --- | --- |
| Mortality |  | Cement | Mud | Cement | Mud |
| 24h | LT90 | - | - | 0.8 | - |
|  | LT50 | 4.3 | 1.9 | 7.5 | 3.6 |
|  | LT10 | 13.9 | 15.3 | 14.1 | 20.0 |
| 48h | LT90 | 1.7 | - | 5.3 | 1.6 |
|  | LT50 | 8.4 | 8.2 | 10.0 | 10.1 |
|  | LT10 | 15.1 | 18.0 | 14.7 | 18.7 |
| 72h | LT90 | 5.0 | 2.8 | 7.7 | 7.8 |
|  | LT50 | 10.6 | 12.0 | 11.5 | 13.6 |
|  | LT10 | 16.1 | 21.2 | 15.4 | 19.5 |
| 96h | LT90 | 7.9 | 6.7 | 9.1 | 9.9 |
|  | LT50 | 12.0 | 13.3 | 13.1 | 16.3 |
|  | LT10 | 16.1 | 19.9 | 17.2 | 22.7 |
| 120h | LT90 | 9.4 | 8.9 | 10.9 | 12.7 |
|  | LT50 | 12.7 | 13.9 | 14.6 | 19.7 |
|  | LT10 | 16.0 | 18.9 | 18.3 | 26.7 |
